# Cloning and Functional Characterization of Putative *Escherichia coli* ABC Multidrug Efflux Transporter YddA

**DOI:** 10.4014/jmb.2003.03003

**Published:** 2020-04-29

**Authors:** Zhenyue Feng, Defu Liu, Ziwen Liu, Yimin Liang, Yanhong Wang, Qingpeng Liu, Zhenhua Liu, Zhongjing Zang, Yudong Cui

**Affiliations:** 1 College of Animal Science and Veterinary Medicine, Heilongjiang Bayi Agricultural University, Daqing 163319, P.R. China; 2 College of Life Science and Technology, Heilongjiang Bayi Agricultural University, Daqing 163319, P.R. China

**Keywords:** Multidrug efflux gene, *yddA*, *Escherichia coli* K-12, ATP-binding cassette, multidrug exporter

## Abstract

A putative multidrug efflux gene, *yddA*, was cloned from the *Escherichia coli* K-12 strain. A drug- sensitive strain of *E. coli* missing the main multidrug efflux pump AcrB was constructed as a host and the *yddA* gene was knocked out in wild-type (WT) and drug-sensitive *E. coli*Δ*acrB* to study the *yddA* function. Sensitivity to different substrates of WT *E.coli*, *E. coli*Δ*yddA*, *E. coli*Δ*acrB* and *E. coli*Δ*acrB*Δ*yddA* strains was compared with minimal inhibitory concentration (MIC) assays and fluorescence tests. MIC assay and fluorescence test results showed that YddA protein was a multidrug efflux pump that exported multiple substrates. Three inhibitors, *ortho*-vanadate, carbonyl cyanide m-chlorophenylhydrazone (CCCP), and reserpine, were used in fluorescence tests. *Ortho*-vanadate and reserpine significantly inhibited the efflux and increased accumulation of ethidium bromide and norfloxacin, while CCCP had no significant effect on YddA-regulated efflux. The results indicated that YddA relies on energy released from ATP hydrolysis to transfer the substrates and YddA is an ABC-type multidrug exporter. Functional study of unknown ATP-binding cassette (ABC) superfamily transporters in the model organism *E. coli* is conducive to discovering new multidrug resistance-reversal targets and providing references for studying other ABC proteins of unknown function.

## Introduction

Bacteria have several efflux pumps associated with drug resistance. By energy source and amino acid homology, drug efflux proteins of bacteria are usually divided into seven superfamilies: the ATP-binding cassette (ABC), the small multidrug resistance (SMR), the major facilitator (MFS), the multidrug and toxic compound extrusion (MATE), the resistance/nodulation/cell division (RND), the proteobacterial antimicrobial compound efflux (PACE) family and the p-aminobenzoyl-glutamate transporter (AbgT) family [1]. Of these, SMR, MFS, MATE, RND, PACE and AbgT use proton kinetic potential as energy and ABC transporters use ATP as energy. All of them can export drugs and other substrates out of cells [2]. Some proteins, although they confer low levels of resistance, are often the first step in resistance, eventually leading to higher resistance by acquiring chromosomal mutations that target antibiotics. The ABC transporter is one of these proteins [3].

ABC transporters exist in almost all organisms from microorganisms to humans [4, 5]. ABC transporters have attracted extensive attention because of their involvement in important physiological processes such as bacterial resistance, human cystic fibrosis, and tumor cell resistance to chemotherapy drugs [6]. ABC transporters contain similar topologies, with two transmembrane domains (TMD) and two nucleotide-binding domains (NBD). These four domains can fuse to form a full transporter such as the multidrug efflux pump P-glycoprotein (P-gp, ABCB1 or MDR1) in humans. In bacteria, multidrug efflux pumps usually fuse to form a half-transporter with a TMD and an NBD domain [7]. The half-transporter forms a homodimer such as MsbA [7] or LmrA [8]. It can also form heterodimers such as EfrCD [9], YheI/YheH [10] and PatA/PatB [11]. The *E. coli* K-12MG1655 strain genome was predicted to encode 69 ABC transporters. Eleven of the ABC transporters are presumed to be exporters [5, 12]. Efflux systems of the ABC family in *E. coli* were reported to mainly include capsular polysaccharide transport pump KpsT [13], hemolysin transport pump HlyA [14, 15], macrocyclic lipid drug efflux pump MacAB [16], outer membrane lipoprotein transport pump LolACDE [17-19], multidrug resistance and phospholipid A transporter MsbA [20], micromycin J25 efflux pump YojI [21], and heme efflux pump CcmABC [22]. Drug efflux systems in *E. coli* that have been reported included MsbA and MacA. MsbA is a multidrug efflux pump and MacAB is a single-drug efflux pump of macrolides[16]. Studies reported on the mechanism of MsbA in multi-drug resistance (MDR), but MDR usually results from a combination of several efflux pumps. Therefore, functional studies on putative ABC efflux pumps in *E. coli* can deepen our understanding of the mechanism of multiple drug resistance. The *E. coli* K-12 genome has five predicted ABC family drug efflux pumps, YddA, YbhFSR, YhiH/YhhJ, YadGH and MdlAB, but relatively few reports of them are available. Amino acid sequence analysis showed that the N-terminus of YddA protein is a transmembrane domain formed by five transmembrane helix domains (TMDs). The C-terminus is a nucleotide-binding domain (NBD), and the NBD domain has Walker A, Walker B, signature, and other important motifs. However, few reports are available on the exact function of YddA protein. If YddA is an efflux pump of the ABC family, whether it can transfer a single substrate or recognizes and transfers multiple substrates at the same time is unclear. Further experiments are needed.

## Materials and Methods

### Bacterial Strains and Plasmids

Bacterial strains and plasmids used in this study are in Table 1. Wild-type (WT) *E. coli, E. coli*Δ*yddA*, *E. coli*Δ*acrB* and *E. coli*Δ*acrB*Δ*yddA* strains were cultured in LB liquid medium at 37°C. A drug-sensitive strain was constructed by knocking out the *acrB* gene in *E. coli* K-12, then the *yddA* gene was knocked out in WT *E. coli* and *E. coli*Δ*acrB*. PCR amplification used pKD3 or pKD4 plasmids as templates. Target fragments and electrocompetent cells with pKD46 plasmid were added to a chamber and subjected to electric shock with a Bio- Rad electroporation system (Bio-Rad, USA). Positive clones from plate were selected for PCR verification (Table S1 and Fig. S1). Assay protocols were described by Datsenko and Wanner [23].

### Chemicals

Tetracycline, oxytetracycline, doxycycline, rifampicin, norfloxacin, cefminox, cefazolin, daunorubicin, doxorubicin, streptomycin, roxithromycin, deoxycholate, chloramphenicol, ofloxacin, acridine flavin, sodium cholate, ampicillin, quinine, Hoechst 33342, and ethidium bromide were from Coolaber (China). *Ortho*-vanadate was from Sigma-Aldrich (USA). Carbonylcyanide m-chlorophenylhydrazone (CCCP) and reserpine were from MedChemexpress (Monmouth Junction, USA). Norfloxacin disks were from Hangzhou Microbial Preparation (China). Gel extraction kits were from ThermoScientific. Wizard SV Gel and PCR Clean-UP System were from Promega (Promega, USA). DH5α competent cells were from Tiangen Biochemical Technology Co., Ltd. (China). Competent cell preparation kits were from TaKaRa (Japan).

### Bioinformatics Analysis of YddA

FASTA format sequences were obtained from the National Center for Biotechnology Information (NCBI) for all proteins. ClustalX software (https://evomics.org/resources/software/bioinformatics-software/clustal-x/) was used for multiple sequence alignments of the YddA and other homologous proteins. Genetic distances were calculated and phylogenetic trees were constructed with Mega 7.0 software (https://www.megasoftware.net/) using the neighbor-joining method. A homology alignment of YddA with other proteins of the ABC family was conducted to identify conserved motifs using DNAStar software (https://www.dnastar.com/). Basic physicochemical properties of the protein were predicted using Protparam software (https://web.expasy.org/protparam/). The transmembrane domain was predicted using TMHMM2.0 software (http://www.cbs.dtu.dk/services/TMHMM/). ProtScale software (https://web.expasy.org/protscale/pscale/Bulkiness.html) was used to analyze hydrophobicity.

### Cloning and Sequencing of *yddA*

The *yddA* sequence was obtained from NCBI. We designed specific primers (*yddA*-F and *yddA*-R, Table S1) for PCR amplification. Genomic DNA of WT *E. coli* was used as a template for PCR amplification of the *yddA* gene. The amplified fragment was cloned into the pMD-18T carrier to transform into *E. coli* DH5α competent cells. Positive clones were screened on ampicillin plates. Positive clones were verified by PCR amplification and sequencing (Fig. S2).

### Drug Susceptibility Assays

Bacterial suspensions were corrected to 0.5 MacFarland units with Mueller Hinton (MH) broth, and after 1:1,000 dilution with MH broth, 100 μl was added to each well of black 96-well plates. Antibacterial drug solutions were separately added. The first to eleventh wells were treated with drug solution at 100 μl per well. In the twelfth well, cells were added as growth controls. Cells were cultured approximately 16-20 h at 37°C, and cell densities were determined using a spectrofluorometer (Infinite M200 PRO; Tecan, USA). The lowest drug concentration that completely inhibited bacterial growth in wells was the MIC. MIC assays were according to Morita *et al.* [24] and Ohene-Agyei *et al*. [25]. Three replicate wells were analyzed for each test, and tests were repeated at least three times. Then we studied the sensitivity of the WT *E. coli* and three mutant strains to norfloxacin using disk diffusion tests.

According to the method of Traub *et al.* and Biswas *et al.* [26-28], WT *E. coli* and three mutant strains were tested for drug sensitivity with norfloxacin susceptibility discs. Cells were cultured in LB solid medium with norfloxacin susceptibility discs and grown at 37°C for 24 h. Diameters of inhibition zones were measured with Vernier calipers.

### Fluorescence Tests

**Ethidium bromide efflux and accumulation assays**. Ethidium bromide (EB) is a substrate widely used with bacterial efflux pumps. It is a probe that can detect and quantify bacterial efflux activity. The difference between EB efflux and accumulation assays was that in efflux assays, EB was added to energy-starved cells for 5 min and then glucose (25 mM) was added. Immediately, EB efflux was detected with a spectrofluorometer (Infinite M200 PRO; Tecan) in real time, at 37ºC, with fluorescence emission recorded every 60 sec for 15 min.

The EB efflux assay was performed on WT *E. coli*, *E. coli* Δ*yddA*, *E. coli* Δ*acrB*, and *E. coli* Δ*acrB*Δ*yddA* strains. Cells were cultured in LB liquid medium to an OD_600_ of about 0.6 at 37°C. Cells were harvested and washed twice with 50 mM KPi buffer (pH 7.0) containing 5 mM MgSO4 and suspended in the same buffer, 0.5 mM 2,4- dinitrophenol was added to cell suspensions for 30 min to consume cellular energy. Cell suspensions were washed three times with 50 mM KPi (pH 7.0) containing 5 mM MgSO4 and 2.5 μM EB was preloaded into the energy-poor cells. Cultures were incubated for 5 min at 37°C, 25 mM glucose was added, and the EB efflux experiment was initiated. Excitation wavelength was 500 nm (9-nm slit), and emission wavelength was 580 nm (20-nm slit). The EB efflux tests were performed according to the protocol as described by Abdel-Motaal *et al*. [29], Reuter *et al*. [8]and Balakrishnan *et al*. [30].

In accumulation assays, WT *E. coli*, *E. coli*Δ*yddA*, *E. coli*Δ*acrB*, and *E. coli* Δ*acrB*Δ*yddA* strains were grown in LB liquid medium supplemented with 0.5 mM 2,4-dinitrophenol. Cells were harvested at mid-logarithmic phase, washed with 50 mM KPi (pH 7.0) buffer containing 5 mM MgSO4 and 25 mM glucose, and suspended in the same buffer. EB (2.5 μM) and inhibitors (*ortho*-vanadate 1 mM, CCCP 40 mM, reserpine 80 μM) were added to cell suspensions. Cells were incubated for 10 min at 37°C, and fluorescence was recorded using a spectrofluorometer (Infinite M200 PRO; Tecan). Excitation and emission wavelengths were 500 nm and 580 nm.

**Norfloxacin accumulation and efflux assays.** WT *E. coli*, *E. coli*Δ*yddA*, *E. coli*Δ*acrB*, and *E. coli*Δ*acrB*Δ*yddA* were grown in liquid LB medium supplemented with 40 mM potassium lactate to OD_600_ 1.0. Cells were collected by centrifugation, washed with 0.1 M Tris-HCl buffer (pH 7.0), and resuspended in the same buffer to OD 600 1.0. After incubation at 25°C for 5 min, norfloxacin (16 μg/ml) was added to initiate the assay. One-milliliter samples were removed at timed intervals and immediately centrifuged at 12,000 ×*g* for 1 min at 4°C and then washed with the same buffer. Pellets were resuspended in 1 ml 100 mM glycine-HCl (pH 3.0). Where indicated, *ortho*-vanadate (1 mM), CCCP (40 μM) or reserpine (80 μM) was added to the assay mixtures. Cell suspensions were shaken vigorously at 37°C for 1 h to lyse cells, centrifuging at 12,000 ×*g* for 5 min at 4°C. The supernatants were diluted 2- fold with the same buffer and fluorescence values detected by a spectrofluorometer (Infinite M200 PRO; Tecan). The excitation and emission wavelengths were 277 and 448 nm for norfloxacin. The norfloxacin accumulation test was performed as described by Abdel-Motaal *et al*. [29].

Norfloxacin is a quinolone antibiotic. Quinolones have strong stable fluorescence in acidic media because they contain conjugated systems of chromophores or cosmophores. Therefore, to detect norfloxacin efflux, we referred to classic efflux detection methods for autofluorescent drugs such as tetracycline [31] or doxorubicin [32], modified according to norfloxacin properties which have higher fluorescence intensity under acidic media.

WT *E. coli*, *E. coli*Δ*yddA*, *E. coli*Δ*acrB*, and *E. coli*Δ*acrB*Δ*yddA* strains were grown in liquid LB medium to OD_600_ 0.8. Cells were resuspended and washed twice with 0.1 M Tris-HCl buffer (pH 7.0) and resuspended in 0.1 M Tris- HCl buffer (pH 3.0.) Norfloxacin (16 μg/ml) was added and cells incubated at 37°C for 5 min. Samples (200 μl) were put into black 96-well plates and glucose was added to cell suspensions (25 mM) to initiate norfloxacin efflux. Efflux of norfloxacin was continuously measured by a spectrofluorometer. The excitation and emission wavelengths were 277 and 448 nm.

**Inhibition of norfloxacin transport by MDR substrates.** To determine if efflux of norfloxacin was inhibited by known MDR substrates, we used WT *E. coli* and mutant strains. The cells were grown to OD_600_ 0.8. Collection of the cells was the same as for the norfloxacin accumulation assays. We mixed five concentrations of norfloxacin (2, 4, 8, 16, and 32 μg/ml) with fixed concentrations of EB (or other MDR substrates) in Mg2+ buffer containing cells. Four concentrations of EB (2.5, 5, 10, and 20 μM) were examined. This method was used to study the inhibitory kinetics of norfloxacin against different drugs [32]. The norfloxacin transport rate obtained with 32 μg/ml norfloxacin and 0 μM EB (or another drug) was 1.0. The relative rates of each efflux curve were calculated, and data were plotted as single-substrate/single-inhibitor kinetics using Origin8.0 software. The IC_50_ value was determined based on the concentration of the drug with a 50% inhibitory effect on 32 μg/ml norfloxacin efflux. Assays were conducted as described by Li *et al*. [32].

**Norfloxacin induction and qRT-PCR analysis.** To explore whether norfloxacin can induce the transcription of *yddA*, WT *E. coli* and *E. coli*ΔacrB strains were incubated in the presence of norfloxacin. Cells were cultured in 2 ml of MH broth medium with low concentrations of norfloxacin. After incubation at 37°C for 12 h, the concentration of selective norfloxacin was increased to continue the next induction. Finally, a series of induced mutants with different MICs of norfloxacin were obtained.

Total RNA was extracted by using the conventional Trizol method. The first strand cDNA was synthesized with a Superscript III reverse transcription kit (Bioneer). The specific primers used for RT-PCR are shown in Table S1. GapA was used as the housekeeping gene. qRT-PCR was performed with a CFX96 fluorescence quantitative PCR instrument (Bio-Rad, USA). The protocol was as described by the LightCycler 480 SYBR Green I Master kits (Roche, Switzerland) The 2^-ΔΔCt^ method was used in the calculations. Three independent qPCR experiments were performed for each sample.

### Statistical Analysis

Experiments were repeated at least three times. Student’s *t*-tests were used to analyze differences among groups. Data are expressed as mean ± standard deviation.

## Results

### The ABC Transporter YddA is Highly Conserved

**Fig. 1:**
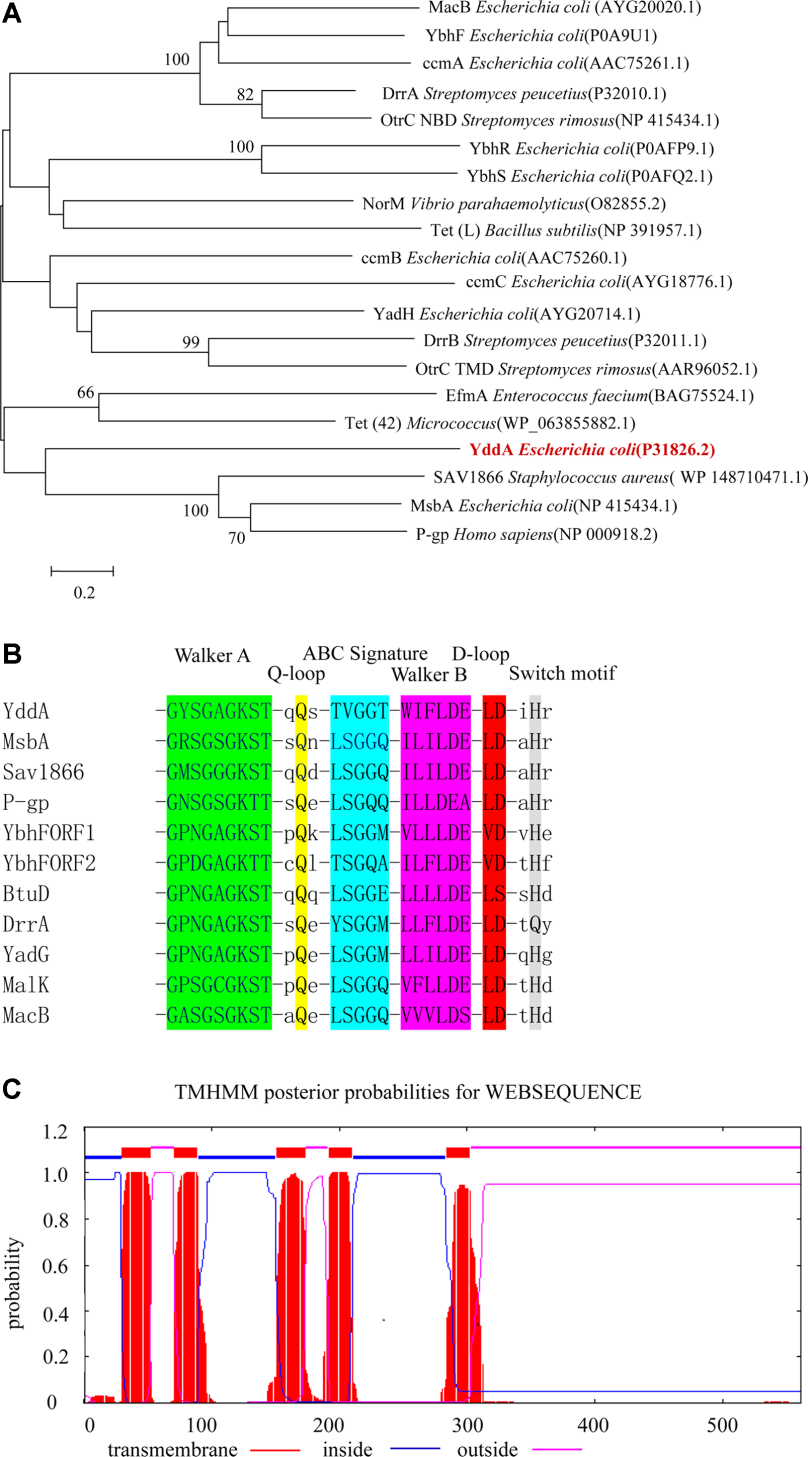
Bioinformatics Analysis of the YddA Protein. (**A**) Phylogenetic tree of YddA and its selected homologs. MEGA7.0 software was used to construct the phylogenetic tree of YddA. Bar, 0.2 substitutions per amino acid residue position. (**B**) Alignment of the amino acid sequence of the YddA region with ABC drug-resistance transporters. There are at least six highly conserved motifs, in the order of Walker A, Q-loop, ABC signature motif, Walker B, D-loop and switch motif. (**C**) The TMHMM2.0 software was used to predict the transmembrane domain at the N-terminus of YddA protein. The results show that the YddA protein encoded five putative α-helical transmembrane segments. Helix positions of TMHMM are located at 30- 52, 72 -94, 151-173, 188-210, 284-306.

Phylogenetic trees were constructed using the adjacency method of MEGA7.0 software of the YddA protein and the reference sequence. Cluster analysis results showed that YddA was first grouped with four MDR efflux pumps are given known ABC family MDR efflux pumps, MsbA (*E. coli*), LmrA (*Lactococcus lactis*), HorA (*Lactobacillus brevis*), and Sav1866 (*Staphylococcus aureus*) (Fig. 1A). This result indicated that YddA was closely related to these three MDR multidrug efflux pumps. The Protparam program was used to predict physical and chemical properties and composition of the amino acid sequence encoded by YddA. The *yddA* gene encodes a protein of 561 amino acids, with a molecular weight of 64.98 kDa. Using DNAstar software, analysis of the C-terminus of YddA showed that YddA contained conserved Walker A, Q-loop, Walker B, ABC signature, D-loop and Switch motifs (Fig. 1B). These motifs were closely associated with ATP binding and hydrolysis, and conserved domain analysis results indicated that YddA had a nucleotide-binding domain (NBD) belonging to the ABC superfamily. The transmembrane domain of YddA was predicted using TMHMM2.0 software. The bioinformatics analysis results preliminarily showed that YddA was similar to MsdA (*E. coli*), P-gp (human) and Sav1866 (*S. aureus*), suggesting that YddA was a putative multidrug efflux pump belonging to the ABC family of *E. coli* (Fig. 1C).

### Cloning of *yddA*

The *yddA* gene was amplified using genomic DNA of *E. coli* K-12 as template, and a 1,686 bp fragment was found by 1% agarose gel electrophoresis, consistent with the expected fragment size. The fragment was recovered and purified and ligated to the pMD18T vector. Single colonies picked from ampicillin-resistance plates were verified by PCR (Fig. S2) and sequencing verification. Results were consistent with NCBI sequences.

### Resistance of YddA Transporter to Drugs

To test the sensitivity of YddA to antibacterial drugs, we tested several classes of antibiotics (beta-lactams, macrolides, aminoglycosides) and other substrates such as EB and Hoechst 33342. WT *E. coli* was the control strain for *E. coli*Δy*ddA* and *E. coli*Δ*acrB* was the control strain for *E. coli*Δ*acrB*Δ*yddA*. Because the multidrug efflux function of AcrB has been studied, the sensitivity of the *acrB* knockout strain *E. coli*Δ*acrB* to different drugs was similar to previous studies. Therefore, we did not discuss the sensitivity of *E. coli*Δ*acrB* strains. The MIC results for 20 substrates showed: (i) When the *yddA* gene was knocked out in WT *E. coli* and *E. coli*Δ*acrB*, three mutant strains showed increased sensitivity to rifampicin, norfloxacin, ofloxacin, tetracycline, oxytetracycline, doxycycline, cefazolin, EB and Hoechst 33342. (ii) When the *yddA* gene was knocked out in *E. coli*Δ*acrB,* cells showed increased sensitivity to cefminox, daunorubicin and doxorubicin. *E. coli*Δ*yddA* cells had the same results as WT *E. coli*. (iii) Three mutant strains showed increased sensitivity to quinine compared to WT *E. coli*. However, this inhibition was different from results in (i), with bacterial growth but densities lower than WT *E. coli* strain. (iv) No difference was seen between the three mutant strains and WT *E. coli* to roxithromycin, chloramphenicol, streptomycin, ampicillin, acridine flavin, sodium cholate, or deoxycholate (Table 2). These results showed that the YddA transporter was a multidrug exporter and had a wide range of substrates.

### Sensitivity of Norfloxacin by Disk Diffusion Test

**Fig. 2:**
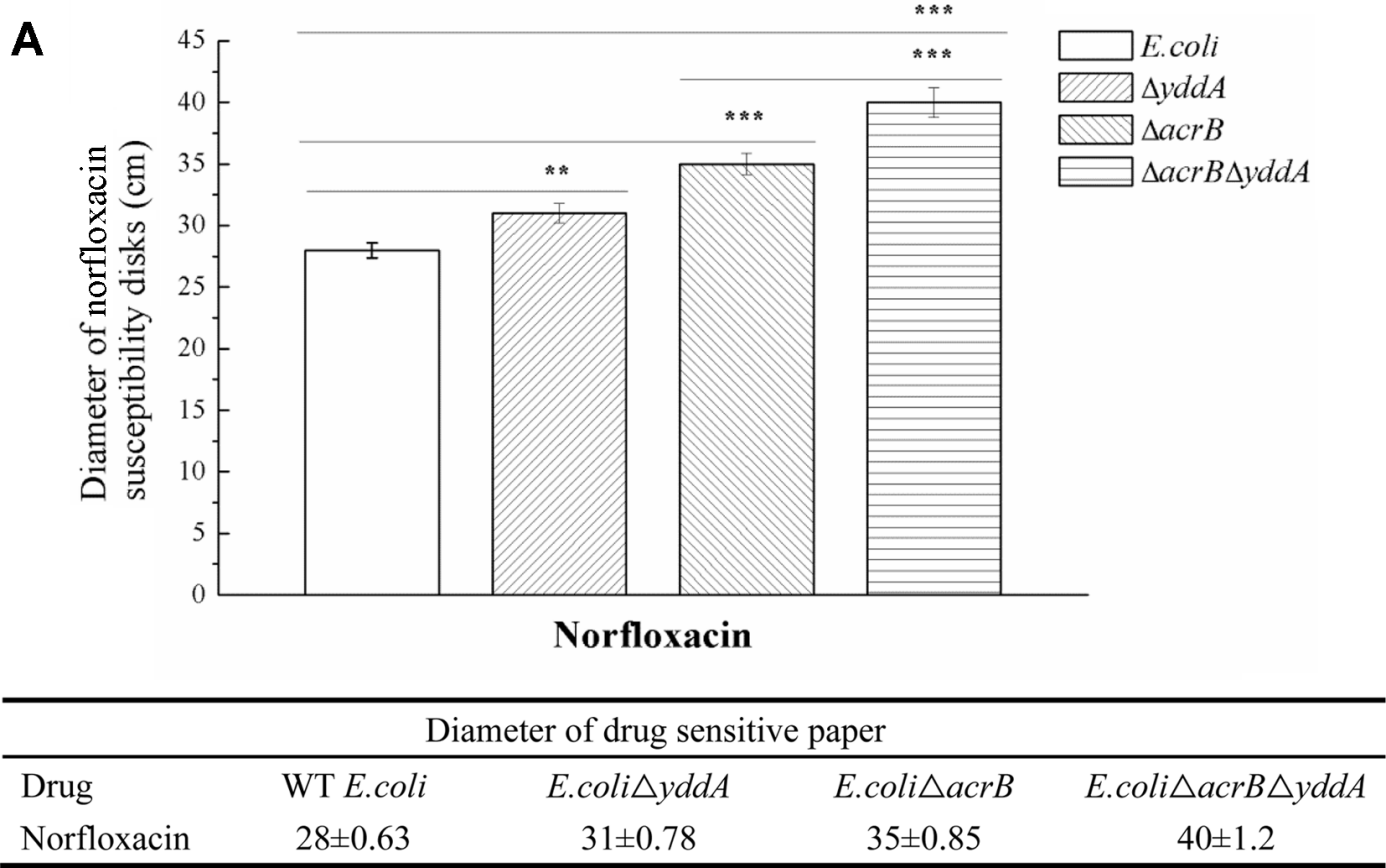
The disk diffusion test. Cells were cultured in LB agar medium, and then coated with drug-sensitive disks, incubated at 37°C for 20 h, and the diameter of inhibition zone was measured with Vernier caliper. The inhibition zone of *E.coli*Δ*acrB*Δ*yddA* was 40 ± 1.2, significantly greater than *E.coli*Δ*acrB*. The inhibition zone of *E.coli*Δ*yddA* (31 ± 0.78) was greater than WT *E. coli* (28 ± 0.63). Statistical significance was: *p < 0.05; **p < 0.01; and ***p < 0.001.

Disk diffusion tests were used to verify results of norfloxacin. Strains showed results consistent with the MIC experiments. The inhibition zone of *E. coli*Δ*acrB*Δ*yddA* was significantly larger than for the *E. coli*Δ*acrB*-sensitive strain, The single-knockout strain *E. coli*Δ*yddA* had increased sensitivity to norfloxacin, as shown by a diameter increase of at least a 5-mm compared to the WT *E.coli*. Inhibition zones for different strains are in Fig. 2. This result indicated that sensitivity to norfloxacin was increased after *yddA* gene was knocked out.

### YddA was an Exporter Involved in EB Extrusion

**Fig. 3:**
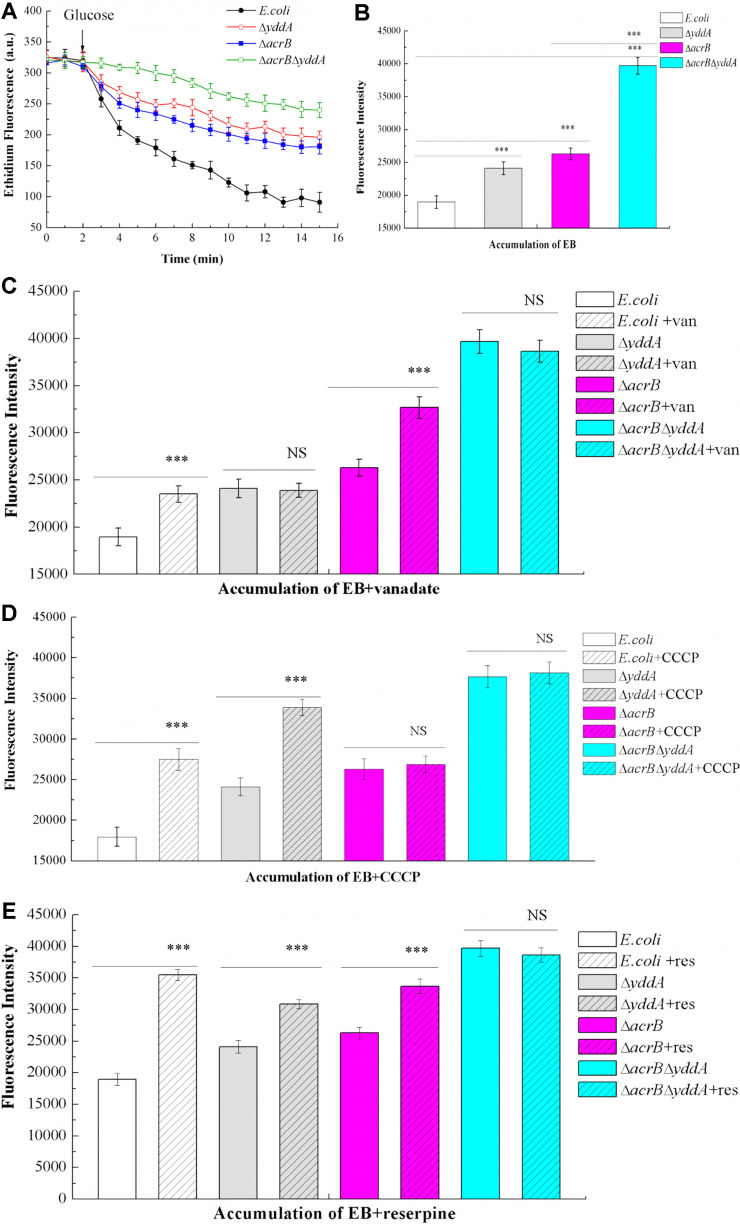
Efflux and accumulation of EB. (**A**) EB efflux. Cells were harvested with 50 mM KPi buffer (pH 7.0), 2.5 μM EB was preloaded in the energy-poor cells. The cultures were then incubated for 5 min at 37°C, then 25 mM glucose was added, the EB efflux experiment was initiated. (**B**) Accumulation of EB. EB (2.5 μM) and glucose (25 mM) were added to cell suspensions. Cells were incubated for 10 min, and fluorescence was recorded using a spectrofluorometer. (**C**) Inhibition of EB accumulation by vanadate. The accumulation of EB in different four strains was detected at the time point of 10 min after adding EB, vanadate and glucose. (**D**) Inhibition of EB accumulation by CCCP. (**E**) Inhibition of EB accumulation by reserpine. Statistical significance was: *p < 0.05; **p < 0.01; and ***p < 0.001.

As shown in Fig. 3A, compared with WT *E. coli* strain, the abilities of *E. coli*ΔacrB, *E. coli*Δ*yddA*, and *E. coli*Δ*acrB*Δ*yddA* to perform EB efflux were significantly lower, and the efflux ability of the *E. coli*Δ*acrB*Δ*yddA* double knockout was the lowest.

The accumulation of EB in four strains was detected at 10 min after adding EB and glucose simultaneously. Three inhibitors (*ortho*-vanadate 1 mM, CCCP 40 μM, reserpine 80 μM) were added to cell suspensions. Consistent with the results of efflux assays, when no inhibitor was added, levels of EB accumulation in the three knockout strains were significantly higher than in the control WT *E. coli*. Levels were higher in double knockout strains *E.coli*Δ*acrB*Δ*yddA* than in *E. coli*Δ*acrB* strains (Fig. 3B), indicating that *yddA* knockout reduced EB efflux, which increased the accumulation of EB in cells. *Ortho*-vanadate is a known ABC protein inhibitor that blocks hydrolysis of ATP and inhibits ATPase activity. To study if EB transport was inhibited by *ortho*-vanadate, we examined the effects of inhibitors in accumulation assays. Fluorescence values for WT *E. coli* and *E. coli*Δ*acrB* cells were increased, but the other two strains *E. coli*Δ*yddA* and *E. coli*Δ*acrB*Δ*yddA* had no change (Fig. 3C). These results were consistent with the bioinformatics analysis and the energy source of the YddA transporter was ATP. CCCP had no significant effect on YddA regular export (Fig. 3D). Reserpine is an inhibitor of multidrug efflux pumps. The most obvious decrease after the addition of reserpine was in WT *E. coli*, followed by *E. coli*Δ*acrB* and *E. coli*Δ*yddA*, whereas the results in *E. coli*Δ*acrB*Δ*yddA* showed no significant decrease in fluorescence (Fig. 3E), indicating that YddA was a multidrug efflux pump and its efflux ability was inhibited by the efflux pump inhibitor reserpine.

### Accumulation and Efflux of Norfloxacin

**Fig. 4:**
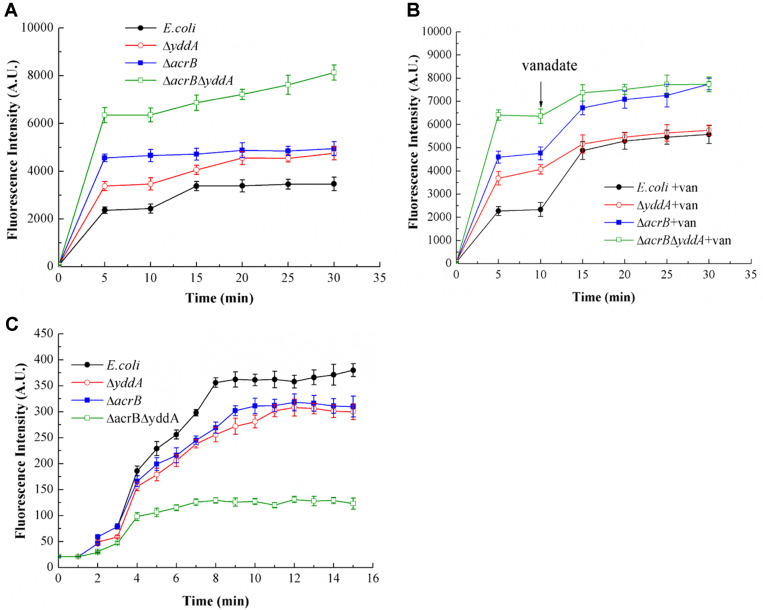
Norfloxacin transport. The fluorescence was detected at excitation of 277 nm (9-mm slit), and emission was monitored at 448 nm (20-mm slit). (**A**) Norfloxacin accumulation. The WT *E. coli* and mutant strains were collected, norfloxacin (16 μg/ml) was added to bacteria suspensions and the assay was initiated. One-milliliter samples were removed at timed intervals and measured using spectrofluorometry. (**B**) Inhibition of Norfloxacin accumulation by vanadate. Norfloxacin (16 μg/ml) and vanadate (1 mM) were added to the assay mixtures to initiate the assay. (**C**) Norfloxacin efflux. Strains were grown in liquid LB medium to OD_600_ 0.8. Norfloxacin (16 μg/ml) was added and cells incubated at 37°C for 5 min. Glucose was added to initiate norfloxacin efflux assay.

When a drug efflux gene was knocked out, increased sensitivity was detected in the knockout strains. We also studied accumulation and efflux of norfloxacin. In accumulation experiments, we tested the fluorescence of norfloxacin in four strains at five time points. We found that the double knockout strain *E.coli* Δ*acrB*Δ*yddA* had the highest accumulation of norfloxacin in cells, followed by the single knockout strain *E. coli*Δ*acrB* and *E. coli*Δ*yddA*. WT *E. coli* had the lowest accumulation of norfloxacin (Fig. 4A). Inhibitor *ortho*-vanadate was used in norfloxacin accumulation tests. We observed that *ortho*-vanadate significantly increased the accumulation of norfloxacin in WT *E. coli* and *E. coli*Δ*acrB* cells (Fig. 4B). In efflux experiments, fluorescence data were acquired every 60 sec for a total of 15 min and efflux of norfloxacin was continuously detected in four strains. The results showed that with increased time, the highest level of increase in fluorescence intensity was detected in WT *E. coli*, followed by single knockout strains *E. coli*Δ*acrB* and *E. coli*Δ*yddA*. Increased fluorescence in double knockout strains was detected, but with the smallest increase of the four strains (Fig. 4C). The results showed that knockout of *yddA* led to less norfloxacin efflux out of cells. Most efflux of norfloxacin was completed in about 10 minutes. These data were consistent with previous reports on efflux time of norfloxacin [33].

### Inhibition of Norfloxacin Efflux by *Ortho*-vanadate and Reserpine

**Fig. 5:**
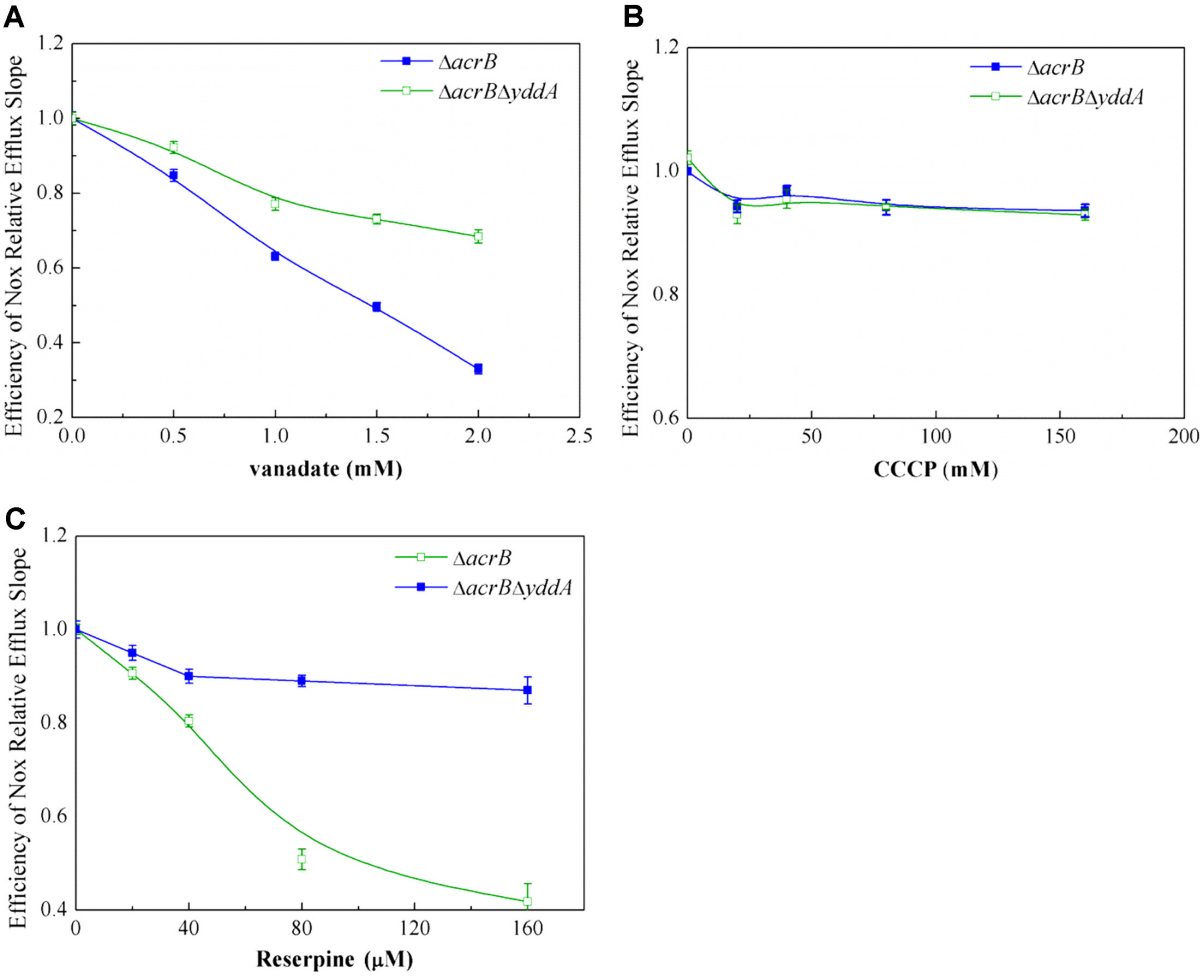
Norfloxacin transport was inhibited by inhibitors. Norfloxacin efflux was measured using whole cells in the presence of norfloxacin (16 μg/ml), and increasing concentrations of the inhibitors in Mg2+ buffer. The slope of the efflux curve obtained at 0 concentration of inhibitor was designated as 1.0. The relative slope of each curve was then determined. (**A**) Kinetic analysis of the inhibitory effect of vanadate on norfloxacin efflux activity. The assay was carried out in the presence of increasing concentrations of vanadate ranging from 0 to 2.0 mM. (**B**) Kinetic analysis of the inhibitory effect of CCCP on norfloxacin efflux activity (CCCP ranging from 0 to 160 μM). (**C**) Kinetic analysis of the inhibitory effect of reserpine on norfloxacin efflux activity (reserpine ranging from 0 to 160 μM).

Inhibitors *ortho*-vanadate, reserpine and CCCP were used in efflux assays of norfloxacin. In whole cells, while maintaining the concentration of norfloxacin (16 μg/ml), the concentration of *ortho*-vanadate (0.5-2 mM) or reserpine (20-160 μM) was increased gradually. *Ortho*-vanadate (Fig. 5A) and reserpine (Fig. 5B) both inhibited the efflux of norfloxacin after *yddA* was knocked out, and the IC_50_ of *ortho*-vanadate was 1,000 μM and the IC_50_ of reserpine was 120 μM. These data are consistent with previous reports on the inhibitory effects of *ortho*-vanadate on DrrAB, P-gp [34] and MsbA [35]. *Ortho*-vanadate is an ATPase inhibitor that inhibits hydrolysis of ATP and substrate efflux. CCCP is an uncoupler of the proton-motive force that had no significant effects on efflux of norfloxacin (Fig. 5C). These results indirectly showed that the energy source of YddA was ATP instead of proton- motive force, consistent with the bioinformatics analysis. Efflux of norfloxacin by YddA transporter was inhibited by reserpine, indicating that YddA was a multidrug efflux pump like other known MDR transporters such as P-gp and MsbA.

### Inhibition of Norfloxacin Efflux by MDR Substrates

**Fig. 6:**
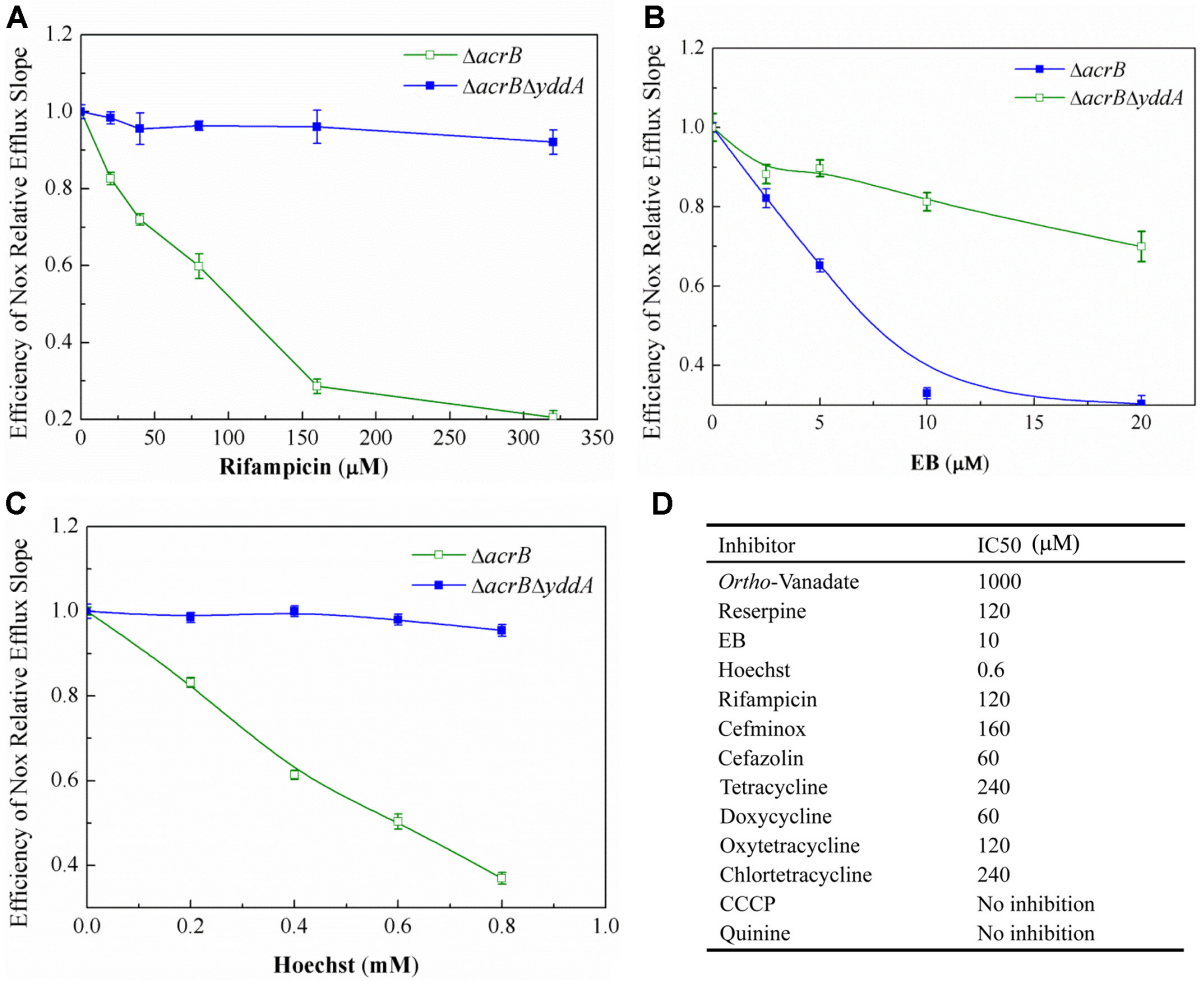
Norfloxacin transport was inhibited by MDR substrates. (**A**) Kinetic analysis of the inhibitory effect of rifampicin on norfloxacin efflux activity (rifampicin ranging from 0 to 325 μM). (**B**) Kinetic analysis of the inhibitory effect of EB on norfloxacin efflux activity (EB ranging from 0 to 20 mM). (**C**) Kinetic analysis of the inhibitory effect of Hoechst33342 on norfloxacin efflux activity (Hoechst33342 ranging from 0 to 0.8 mM). (**D**) A table showing a summary of the IC50 values.The IC50 values were calculated as described under “Materials and Methods.”

To study if the YddA transporter recognized and bound other known MDR substrates, the inhibitory effect of different drugs on norfloxacin efflux was studied in whole cells. Measurements were taken at multiple inhibitor concentrations while maintaining a constant norfloxacin concentration. Some MDR substrates including rifampicin (Fig. 6A), EB (Fig. 6B), Hoechst 33342 (Fig. 6C), cefminox, cefazolin, tetracycline, doxycycline, oxytetracycline and chlortetracycline significantly inhibited YddA-mediated norfloxacin efflux. CCCP were unable to inhibit norfloxacin efflux. Fig. 6D summarizes the inhibitory effects of several MDR substrates. The IC_50_ values of substrates differed widely. For example, the IC_50_ for EB and Hoechst33342 was relatively low, while the IC_50_ for cefminox, cefazolin, tetracycline, doxycycline, oxytetracycline, chlortetracycline and rifampin was higher to achieve the same level of inhibition. These results indicated that the YddA transporter had multiple substrates with different affinities. However, whether these drugs are bound to the same site or have multiple drug-binding sites cannot be determined from these data.

### Norfloxacin Upregulates the Transcription of *yddA*

**Fig. 7:**
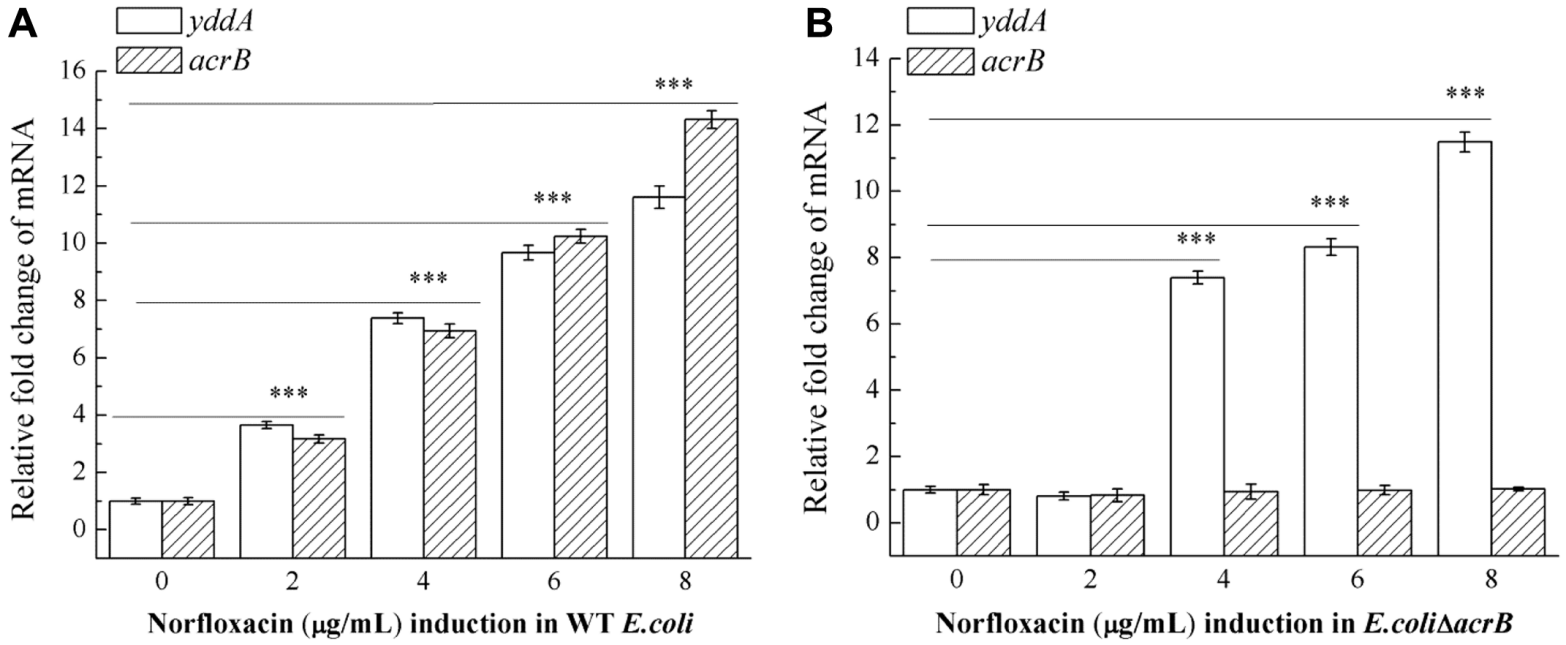
qRT-PCR analysis of the transcriptional level of related genes in norfloxacin-induced resistant strains. Bacteria were grown in LB medium while adding low concentration of norfloxacin for 12 h, finally, a series of induced mutants with different MICs of tetracycline were obtained. Extraction of total RNA from norfloxacin-resistant strains by trizol method, qRT-PCR was performed with a CFX96 fluorescence quantitative PCR instrument. (**A**) The transcriptional level of *yddA* and *acrB* in WT *E. coli*. (**B**) The transcriptional level of *yddA* and *acrB* in *E.coli*Δ*acrB*.

After the induction of increasing concentrations of norfloxacin in WT *E. coli* and *E. coli*ΔacrB, the MIC values of the strains gradually changed, and the expression of *yddA* gene and *acrB* were detected. Both WT *E. coli* and *E. coli*ΔacrB induced strains showed increased MICs for norfloxacin of 8 μg/ml. We studied the relative expression levels of *yddA* and *acrB* genes with real-time PCR in norfloxacin-induced strains WT *E. coli* and *E. coli*Δ*acrB*. The results showed that under norfloxacin induction, the expression levels of *yddA* and *acrB* in WT *E. coli* strain increased gradually with the increase of norfloxacin (Fig. 7A). In the *E. coli*ΔacrB induced strain, almost no *acrB* gene was detected, and the relative transcription levels of *yddA* increased in a stepwise manner (Fig. 7B). Transcription of the *yddA* gene was enhanced when subjected to the norfloxacin stress conditions, suggesting that *yddA* can respond to the norfloxacin exposure, consistent with the predicted role of *yddA* in norfloxacin resistance.

## Discussion

Because of the high redundancy of proton- or ATP-dependent drug transporters and overlapping specificity in the bacterial genome, sequence analysis shows the prevalence of putative MDR pumps. In some bacterial genomes, for instance *Bacillus subtilis*, *Staphylococcus aureus* and *E. coli* may carry up to 30 putative drug efflux pumps[36]. However, many of these systems have not yet been linked to multidrug resistance in bacteria. In eukaryotes, multidrug ABC-type efflux pumps have been well described. For example, P-gp in humans is a typical eukaryotic ABC family protein and is responsible for the MDR phenotype of many cancer cells. Its overexpression correlates with resistance to chemotherapy in patients with tumors [37]. ABC transporters related to drug efflux have also been reported in prokaryotes. Gram-positive bacteria have been identified as having ABC multidrug efflux pumps including HorA [38], LmrA [39], LmrCD [40], BmrCD [41-43], patAB [44], DrrAB [8], EfrCD [9], EfrAB [45, 46], BmrA [47-49], VltAB [27], and MsrA [50]. Most efflux pumps described are driven by protons and few multidrug ABC transporters have been reported in gram-negative bacteria. Multidrug efflux pumps of the ABC family reported in gram-negative bacteria include MsbA [7], MacAB [16, 51], TmrAB [52], VcaM [53], SmrA [54], SmdAB [55]. Compared with functional studies of eukaryotic ABC transporters, the role of bacterial ABC family transporters in drug resistance may be underestimated [56].

In 1997, the genome of the *E. coli* K-12 strain was sequenced [12]. The largest protein family in the *E. coli* K-12 strain is the ATP-binding cassette family. From these genomic data, all encoded ABC proteins were analyzed. Seven of them were putative drug export transporters, with MsbA [7] and MacB having been confirmed as such. MsbA is a multidrug efflux pump and lipid A transporter, and MacB is a single drug efflux pump that can transfer macrolide drugs [16]. Functions of the other five, YddA, MdlAB, YbjYZ, YojHI, YbhFSR, have been only partially reported.

The hallmark of ABC transporters are similar topologies, two transmembrane domains (TMDs) and two cytoplasmic nucleotide-binding domains (NBDs). In bacteria, usually a TMD and an NBD fused to form a semi- transporter. The semi-transporter can form homodimers or heterodimers. The putative drug exporter YddA protein contains an N-terminal transmembrane domain and a C-terminal ATP binding domain to form a half- transporter. This structure is the same as the ABC family of multidrug efflux pumps LmrA (*Lactococcus lactis*), MsbA (*E. coli*), MacB (*E. coli*), and BmrA (*B. subtilis*) [47]. These four known multidrug efflux proteins all function as homodimers. Whether YddA also forms homodimers like these four proteins is unknown, but analysis of the operon in which YddA is located did not find other ABC proteins, and YddA protein should also form homodimers.

In 2001, Kunihiko Nishino and Akihito Yamaguchi analyzed all putative MDR proteins of the *E. coli* genome [57], including the YddA protein. The YddA protein was linked to a vector and expressed in the sensitive strain *E. coli* KAM3 to detect its drug sensitivity. The MIC was tested and no significant increase was seen after YddA overexpression. The methods for identifying ABC-type MDR transporters include knocking out candidate genes that will increase drug sensitivity, and overexpression of MDR transporters in sensitive strains that will increase drug resistance. In our study, *acrB* gene in the AcrAB system was knocked out, disrupting the integrity of the AcrAB transporter and destroying its function [58], and a drug-sensitive strain of *E. coli*Δ*acrB* was constructed. We knocked out of the *yddA* gene in *E. coli*Δ*acrB* and *E. coli* K-12 to study its function and we tested the sensitivity of the four strains to 20 drugs. The MIC assay confirmed that *E. coli*Δy*ddA*, *E. coli*Δ*acrB* and *E. coli*Δ*acrB*Δ*yddA* cells showed increased sensitivity to rifampicin, norfloxacin, ofloxacin, tetracycline, oxytetracycline, doxycycline, cefazolin, EB, Hoechst 33342 and quinine. But there are three drugs, cefminox, daunorubicin and doxorubicinhat that show increased sensitivity only in double knockout strain *E. coli*Δ*acrB*Δ*yddA.* Resistance phenotypes are not easily observable due to the high redundancy of drug transporters with overlapping specificities in bacterial genomes, but studying the function of the target gene in sensitive strains helps. One special drug in the YddA- regulated export, quinine, behaves differently from other drugs in the MIC assay. When quinine was added, the mutant strains *E. coli*Δ*yddA*, *E. coli*Δ*acrB* and *E. coli*Δ*acrB*Δ*yddA* were at a sub-suppressive level. But quinine could not inhibit YddA-regulated norfloxacin efflux. These two results seem contradictory, but they agreed with each other. At the same time, it provided us with a new idea that the efflux of quinine regulated by YddA transporter was not the same as the other substrates, therefore it is possible that the binding site of quinine to the YddA transporter is worse than other substrates.

The drug efflux transporters of ABC family are rarely expressed without performing certain functions; therefore, the expression of genes in the presence of a drug can be analyzed by qRT-PCR assay. If one or more drugs are capable of increasing the expression of a particular ABC gene, it is reasonable to assume that one or more drugs are efflux substrates. Similarly, comparing gene expression between drug-resistant strains and wild-type strains is also useful for identifying proteins associated with drug resistance. Therefore, we used the substrate norfloxacin of YddA transporter, and then tested the transcription levels of related genes in the drug-resistant strains and control strains. The relative expression levels of *yddA* and *acrB* genes with real-time PCR in norfloxacin-induced strains WT *E. coli* and *E. coli*Δ*acrB* was studied. qRT-PCR analysis showed that the transcription of *yddA* was upregulated upon exposure to norfloxacin.

We further detected the efflux and accumulation in cells of fluorescent substrates (EB, norfloxacin). Intracellular fluorescent substrate accumulation was significantly higher for *E. coli*Δ*acrB*Δ*yddA* than *E. coli*Δ*acrB*, while efflux capacity was significantly lower, indicating that *yddA* knockout reduced the efflux capacity and increased the accumulation of the drug. Three inhibitors, *ortho*-vanadate, reserpine, and CCCP were used in fluorescence experiments. *Ortho*-vanadate and reserpine significantly inhibited efflux and increased accumulation of EB, and norfloxacin inhibition was not seen with CCCP. This result showed that the energy source of YddA was ATP instead of proton-motive force. We used known MDR substrates to detect their effect on norfloxacin export. Several MDR substrates, EB, Hoechst 33342, rifampicin, cefminox, cefazolin, tetracycline, doxycycline, oxytetracycline, and chlortetracycline significantly inhibited norfloxacin efflux. Among these substrates, only CCCP and quinine did not inhibit norfloxacin efflux. These results indicated that YddA was a multidrug efflux pump belonging to the ABC family, with a wide range of substrates. Our study on the *yddA* gene is relatively preliminary. In theory, the YddA transporter can only extrude substrates to the periplasmic space instead of extracellular. Other auxiliary proteins are required to complete transportion, such as the membrane fusion protein (MFP) family proteins and the outer membrane channel proteins. Only two genes, *yddA* and *yddB,* were found in the operon where YddA is located. Bioinformatics analysis found amino acids 49-165 of the YddB protein contain a plug domain that belongs to TonB-dependent porin protein and YddB localizes to outer membrane. Some drug efflux transporters in bacterial can form tripartite efflux pumps, and these pumps typically consist of three essential components, an inner membrane transporter, a membrane fusion protein (MFP) and an outer membrane factor (OMF), such as MacAB-TolC system and AcrAB-TolC system from *E. coli*. In contrast, the pumps that are not organized in this manner and exist as single-component pumps in the inner membrane. These single-component pumps may take up drugs from the cytosol and function with porins or other types of protein channels to make the efflux process effective, such as Tet pumps. *YddA* gene is located in an operon with the outer membrane channel protein YddB but not with the MFP protein. Therefore, the YddA protein very likely forms single-component pumps with protein channels YddB to extrude substrates. YddA are similar to some known ABC multidrug transporters but have their own characteristics. The study of YddA protein will increase our understanding of the ABC family.

With the discovery of LmrA, the multidrug resistance of prokaryotes and eukaryotes merged. Prokaryotic multidrug efflux pumps of ABC such as LmrA, MsbA, and Sav1866 [59]were considered structural and functional homologs with P-gp. The ABC family contains thousands of members, most of which are transporters. In bacteria, they participate in the efflux or import of substrates including ions, sugars, lipids, peptides or complex organic molecules. We report here the functional properties of an MDR ABC transporter YddA from the *E. coli*. In our studies we found that YddA transporter had a wide range of drug substrates including tetracyclines (tetracycline, oxytetracycline and doxycycline), quinolones (norfloxacin and ofloxacin), cephalosporins (cefminox and cefazolin), anticancer antibiotics (daunorubicin and doxorubicin), antimalarial drugs (quinine), antituberculosis drugs (rifampicin) and other cationic substrates such as EB and Hoechst 33342. YddA is a prokaryotic homolog of P-gp similar to LmrA, MsbA and Sav1866.

Because multidrug ABC proteins cause serious public health problems, they are a main target of clinical research. In bacteria, several ABC-type multidrug transporters have been discovered, especially in gram-positive bacteria. These include LmrA (*L. lactis*), Sav1866 (*S. aureus*) and BmrA (*B. subtilis*), all of which are related to bacterial resistance. Therefore, the importance of ABC transporters in cell physiology and medicine cannot be underestimated. ABC transporters are now recognized as the most important of all protein families. Research on ABC proteins in various forms has now become a small industry. As in many areas, research on “advanced” eukaryotic systems cannot proceed quickly without fundamental advances in microbial research [34]. YddA is a prokaryotic homolog of P-gp similar to LmrA, MsbA and Sav1866. Structural similarity between YddA and P-gp translates into functional similarity. Both proteins mediate the extrusion of multidrugs and the activities of both transporters were reversed by *ortho*-vanadate and reserpine. Therefore, research on the function of YddA, a putative drug efflux pump of the ABC family of *E. coli*, a model organism of prokaryotes, may provide a useful reference for explaining medically important homologous proteins such as P-gp in humans and pathogenic microorganisms. Understanding the mechanisms by which eukaryotic and prokaryotic cells develop resistance is key to developing effective new drugs. In cells, the multidrug-resistant phenotype often results from multiple mechanisms, which may include multiple transporter systems. Therefore, more research on the function of hypothetical drug efflux pumps will deepen our understanding of the mechanism of MDR transporters. YddA is a multi-drug efflux transporter belonging to ABC superfamily, which can recognize and transport a variety of substrates. Functional studies of the *yddA* gene provide theoretical support for further understanding of the ABC family.

## Supplemental Materials



Supplementary data for this paper are available on-line only at http://jmb.or.kr.

## Figures and Tables

**Table 1:** Bacterial strains or plasmids.

Plasmid	Characteristic(s)^a^	Reference or source
pMD18-T	Cloning vector	Purchased from TaKaRa
pKD46	bla (Ap ^r^), a helper plasmid for expression of Redλ recombinase	Donated by Guoqiang Zhu
pKD3	Template plasmid for a chloramphenicol-resistance gene	Donated by Guoqiang Zhu
pKD4	bla (Ap ^r^ ), a plasmid carrying FRT-flanked adjacent kan (Km ^r^ ) gene	Donated by Guoqiang Zhu
pCP20	bla (Ap ^r^) cat (Cm ^r^), a plasmid for expression of FLP recombinase	Donated by Guoqiang Zhu

Bacterial strains		

*E. coli* K-12	Wild type	Haerbin Veterinary Research Institute
*E. coli* DH5α	△(*arg*F-*lac*169)80*dlac*Z58(M15)*gln*V44(AS)^-^ *rfb*D1*gyr*A96 recA1	purchased from Tiangen
*E. coli*/pMD-18T	clone vector	This work
*E. coli*Δ*acrB*	*E. coli* Δ*acrB*:: Ap ^r^	This work
*E.coli* Δ*acrB*Δ*yddA*	*E. coli* Δ*acrB*Δ*yddA*::Cl ^r^	This work

**Table 2:** Minimal inhibition concentrations (MICs) of tested antimicrobial drugs.

Drugs	Minimum inhibitory concentrations (µg/mL)			

	WT *E. coli*	△*yddA*	△*acrB*	△*acrB*△*yddA*
Rifampicin	16	8	8	2
Norfloxacin	2	0.5	0.125	0.06
Tetracycline	1	0.5	0.25	0.125
Oxytetracycline	32	16	8	4
Doxycycline	32	2	0.125	<0.06
Cefminox	1	1	0.125	<0.06
Cefazolin	64	32	16	2
EB	>128	128	128	16
Hoechst33342	128	64	64	32
Daunorubicin	64	64	1	<0.06
Doxorubicin	4	4	1	0.125
Quinine	>128	16	0.5	0.125
Roxithromyci	>128	>128	>128	>128
Chloramphenicol	8	8	0.5	0.5
Streptomycin	>128	>128	>128	>128
Ampicillin	8	8	4	4
Acridine flavin	0.5	0.5	0.5	0.5
Ofloxacin	0.125	0.06	0.06	<0.06
Sodium cholate	>128	>128	>128	>128
Deoxycholate	>128	>128	>128	>128
