# Supplementary material for: Cloning and Functional Characterization of Putative Escherichia coli ABC Multidrug Efflux Transporter YddA
Source: J Microbiol Biotechnol. 2020 Apr 29;30(7):982–95. doi: 10.4014/jmb.2003.03003 (PMC9728188; doi:10.4014/jmb.2003.03003)
Supplement: Supplementary file 1 [file JMB-30-7-982-supple.pdf]

J. Microbiol. Biotechnol.
https://doi.org/10.4014/jmb.1907.07030

Supplementary Material

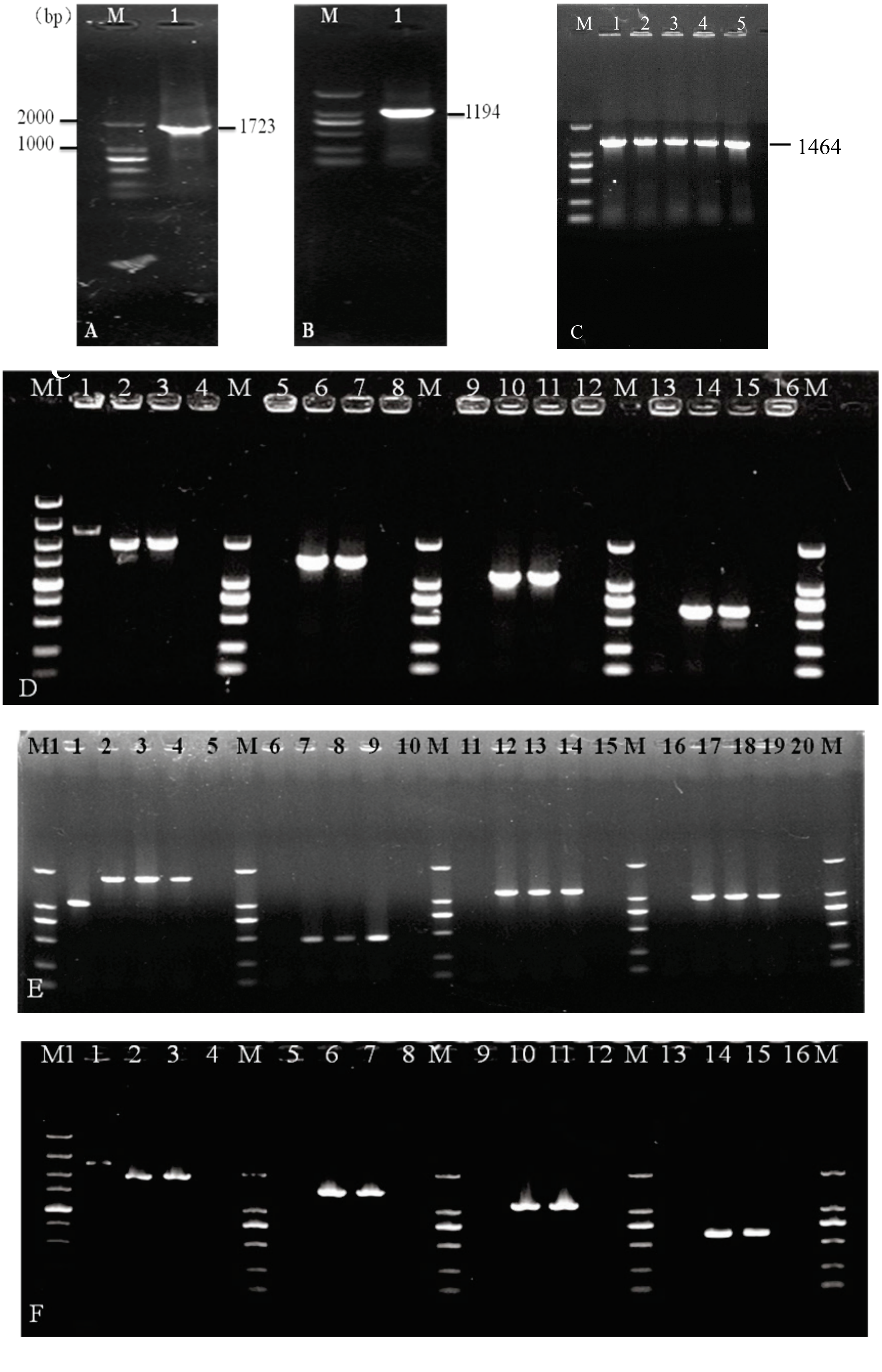

Figure 1. Knock out of genes. M.DL2000. M1.DL5000. (A) Target fragment of *acrB*(1723 bp). (B) Target fragment of *yddA*(1194bp). (C) PCR verification of the pKD46 plasmid was transformed into *E. coli* K-12. M.DL2000 M1.DL5000 Marker,1.pKD46(1464bp). 1-5. Positive clones. (D)Results of positive clones by PCR of Δ*yddA* in *E. coli* K-12. (E)Results of positive clones by PCR of Δ*acrB* in *E. coli* K-12. (E)Results of positive clones by PCR of Δ*yddA* in *E. coli* Δ*acrB*.(F) Growth curve of the recombinant. Take each time point of liquid measuring OD value of 600 nm, draw the growth curve of these strains.

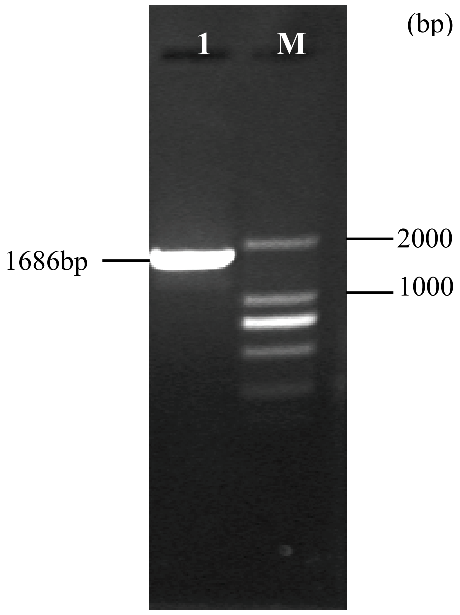

Fig 2. Cloning of *yddA*. M.DL2000. PCR identification of *yddA* was cloned in *E. coli* K-12 with pMD-18T. 1. PCR verification of the *yddA* gene use plasmid as template.

Table 1. Primers used in this paper

primer	Sequence(5'to3')	Size(bp)
yddA-F	CGC<u>GGATCC</u> ATGATAACCATTCCCATTACGCT	32
yddA-R	GG<u>GAGCTC</u>TTATAAAACCGCGCTAATGTCAC	31
pKD46-F	AGAGCTGGGCGCGTCACTAC	20
pKD46-R	TCGTACTGTTTTCCCCAGGC	20
K-*acrB* F	GGATACCGCTGCGGCAATCCGTGCTGAACTGGCGAAGATGGAACCGTGTAG GCTGGAGCTGCTTC	71
K-*acrB* R	CAACGTTGTTCTTTTCTTTGGTCAGATAGTAATGCGTTACCTCATCATATGAA TATCCTCCTTAG	66
K-*yddA*- F	ATGATAACCATTCCCATTACGCTTCGTATGTTGATAGCAAAGTATGTGTAGGC	65

	TGGAGCTGCTTC	
K-*yddA*- R	TTATAAAACCGCGCTAATGTCACAAATATCATCGGCCAGGTTCCACATATGA ATATCCTCCTTAG	65
T-*acrB*- F	GAACTACGACATCATCGCAGAG	22
T-*acrB*- R	TCAAGGAAACGAACGCAATAC	21
T-*yddA*-F	CGTACTTTCTGGCTTGATGT	20
T-*yddA*-R	TTACATTGAAAACTGGAGGG	20
Cm-F	TGAAACTCACCCAGGGATTG	20
Cm-R	ATAAATCCTGGTGTCCCTGT	20
Kan-F	CAGTCATAGCCGAATAGCCT	20
Kan-R	CGGCCACAGTCGATGAATCC	20
RT-*gapA* F	CTGGTGCGAAGAAAGTGGTT	20
RT-*gapA* R	TGATGCCGAAGTTATCGTTG	20
RT- *acrB* F	CGATAACCTGATGTACATGTCC	22
RT- *acrB* R	CCGACAACCATCAGGAAGCT	20
RT-*yddA* F	GCAGTTTATTAGCGGGCAGA	20
RT-*yddA* R	CCAGTTCAGCAAGTTCGTCA	20
